# Is parental anxiety related to child anxiety? Insights from a four-wave longitudinal study in a Chinese context

**DOI:** 10.3389/fpsyt.2025.1570652

**Published:** 2025-04-28

**Authors:** Daniel T. L. Shek, Xiang Li, Banglin Yang, Jiangfeng Yang

**Affiliations:** Department of Applied Social Sciences, The Hong Kong Polytechnic University, Hong Kong, China

**Keywords:** parental anxiety, child anxiety, gender, children, adolescents, longitudinal data

## Abstract

**Objective:**

There has been a growing concern regarding the development of parental anxiety and child anxiety. However, the dynamic bidirectional relationship between parental anxiety and child anxiety remains unclear, particularly across different genders and developmental stages. This study investigated the bidirectional relationships between parental anxiety and child anxiety, and further explored the relationships across gender and age.

**Methods:**

Data were collected across four waves from 2019 to 2022 in Sichuan Province, China, including 6,117 students (49.00% girls; 61.10% adolescents; *M* = 10.32 years, *SD* = 2.14). Parental anxiety was evaluated by the Self-Rating Anxiety Scale (SAS), and child anxiety was assessed using the Screen for Child Anxiety-Related Emotional Disorders (SCARED). A random intercept cross-lagged panel model (RI-CLPM) was constructed to distinguish between within- and between-person levels of parental anxiety and child anxiety.

**Results:**

Results found that parental anxiety significantly predicted child anxiety across all time points, which supports the “parent effects” model. Meanwhile, child anxiety also influenced parental anxiety from Time 3 to Time 4, partially supporting the “reciprocal effects” model. Moreover, the impact of parental anxiety on girls’ anxiety was significantly stronger than on boys, and parental anxiety had a more substantial influence on children than on adolescents.

**Conclusion:**

These findings underscore the distinct roles of gender and developmental stages in the transmission of parental anxiety to children. The present findings provide theoretical and practical evidence for the development of parental anxiety and child anxiety in the Chinese context.

## 1 Introduction

Anxiety is one of the most prevalent emotional issues affecting children and adolescents, with a global prevalence of 6.5% (1). Two meta-analyses showed that the prevalence of anxiety disorders among Chinese children and adolescents was 12.3% and 27%, respectively (2, 3). Furthermore, anxiety is frequently accompanied by other emotional and behavioural issues, such as depression (4), eating disorders (5), internet addiction (6), and sleep disorders (7). In this context, research into the potential mechanisms underlying anxiety among children and adolescents has become extremely important. Among these potential causes, parental anxiety is particularly relevant, as it is shaped by personal experiences and the evolving demands of modern society. For instance, in the context of the “double reduction” policy, parents’ anxiety has become more diverse and is increasing in China (8).

### 1.1 Parental anxiety and child anxiety

Attachment Theory suggests that parent-child attachment shapes children’s internal working model of perceiving and evaluating themselves and others (9–11). In contrast, insecure attachment can lead to the negative internal working model among adolescents, which can result in negative psychological representations of self and others, and leading to the development of negative emotions, cognitions, and behaviors such as anxiety and depression (12, 13). While many studies have explored the relationship between parental anxiety and child anxiety (14, 15), most empirical research only focuses on how parental anxiety affects children’s anxiety (i.e., “parent effect” model) (16, 17). For instance, Ranney et al. (18) revealed that parents’ (both mother and father) anxiety at child age 7 was related to girls’ anxiety at age 15. However, as the family is a dynamic structure where family members influence each other (19), children can also influence parents, (i.e., “child effect” model) (20). The “child effect” model assumes that children’s emotional and behavioral characteristics have a major influence on parents’ behavior (21, 22). For example, a study on genetic sensitivity found that children’s anxiety symptoms at age 7 predicted and increased adoptive mothers’ anxiety symptoms (23). Similarly, a cognitive behavioral therapy (CBT) study found that children’s anxiety symptoms improved after receiving CBT, which also positively impacted parent anxiety and psychological control 12 months later (24).

Additionally, the “reciprocal effects” model assumes the bidirectional of effects in which parents and children contribute to each other’s behavior developmental outcomes (25, 26). Several studies have explored this bidirectional relationship. For instance, Johnco (27) found that parental anxiety significantly predicted adolescent anxiety, whereas adolescent anxiety did not predict parental anxiety, thus only supporting the “parent effect” model. Liu et al. (28) revealed that children showed more anxiety symptoms when their parents reported higher anxiety levels, and children anxiety predicted mothers’ anxiety symptoms in the future.

In addition, parent-child relationships are facing new challenges, such as children’s exposure to digital technology, which exacerbates parental anxiety (29). Parental anxiety has a profound impact on children’s mental health through intergenerational transmission. Therefore, in this context, in-depth research on the intergenerational transmission mechanism of anxiety is of great value for understanding parent-child interaction patterns and optimizing parent-child relationships.

### 1.2 Developmental differences

According to the Social Role Theory, society has different expectations for boys’ and girls’ behaviors and emotions (30). Boys are often viewed as resilient and able to avoid negative emotions (31). In contrast, girls are judged to be more culturally acceptable to express negative emotions (32). Meanwhile, girls are generally more sensitive to emotional cues and are likely to be affected by other people’s emotions (31). Thus, girls may be more affected by parental anxiety (33). Empirical studies have shown that parental anxiety at child age 7 was associated with girls’ anxiety at age 15, but not boys’ anxiety (18). Additionally, traditional Chinese culture places higher expectations for boys, particularly when handling difficulties and assuming responsibilities (34, 35). Hence, parents may perceive boys’ anxiety as a violation of gender role expectations, which lead to anxiety about boys’ ability to handle future responsibilities. In contrast, girls are frequently seen as more emotionally sensitive in traditional Chinese culture, and their anxiety is seen as a more “normal” or “acceptable” trait (36). Therefore, parents may be more accepting and tolerant of girls’ anxiety.

Considering the developmental differences between children and adolescents (37), this study further explores how parental anxiety impacts these two age groups differently. During childhood, parent-child relationship tends to be more harmonious, with children being emotionally dependent on their parents and highly sensitive to emotional responses (38). Children often learn emotional coping strategies by observing and imitating their parents’ behaviors (38). As a result, parental anxiety is more likely to influence children through emotional transmission. However, adolescents develop more sophisticated emotional regulation strategies as they mature psychologically (39), which enables them to cope with negative emotions more effectively, thereby reducing the direct impact of parental anxiety (40). Therefore, parental anxiety is more likely to trigger anxiety in children than in adolescents. In addition, child and adolescent anxiety may affect parental anxiety differently. Children typically share a closer emotional bond with parents, especially when the parents are primary caregivers, which enhances the direct transmission of anxiety (41). As adolescents become more independent, parental influence gradually diminishes (42). Adolescent anxiety tends to manifest more as internalized problems, such as depression and low mood (43), which are less visible to parents. Thus, the effect of adolescent anxiety on parental anxiety may be weaker compared to child anxiety. In the existing scientific literature, 10 years of age is commonly cited as the transition point between childhood and adolescence in various developmental models. For example, Sawyer et al. (44) describe adolescence as running from the age of 10 to 24, emphasizing that the age of 10 marks the beginning of this critical developmental stage.

### 1.3 Research gaps in the current literature

Although many studies have examined the relationship between parental anxiety and child anxiety from different perspectives, the latest scientific literature has many limitations. First, there is a severe lack of research in non-Western contexts, such as China (45). With existing research primarily focused on Western nations (27, 46, 47). In China, many families prioritize submission to parental authority and often promote parenting styles characterized by behavioral control (48–50). These parenting styles may exacerbate the transmission of anxiety from parents to children (50, 51). Hence, it is necessary to investigate the relationship between parental anxiety and child anxiety within the Chinese context. Second, although some studies have explored the longitudinal relationship between parental anxiety and child anxiety (52, 53), few studies distinguish the within- and between-individual levels of parental anxiety and child anxiety. Hence, this study utilized a random intercept cross-lagged panel model (RI-CLPM) to mitigate confounding effects of parental anxiety on child anxiety at both the within- and between-individual levels (54, 55).

Third, existing research has a limited focus on gender, and its findings are inconsistent. In the context of Chinese culture, the impact of gender differences may be particularly pronounced, as girls may be more likely to exhibit anxious behaviors (56, 57). Therefore, the present study further explored the effects of parental anxiety on the anxiety of children of different genders. Fourth, previous research has rarely addressed developmental differences in the intergenerational transmission of anxiety, although some studies have indicated that this transmission may be influenced by timing. For instance, Chen et al. (58) identified infancy and early adolescence as critical developmental periods during which children are particularly vulnerable to the effects of parental anxiety. Additionally, research has shown that anxiety disorders are most prevalent in childhood (59). These findings suggest that children are especially susceptible to parental anxiety.

### 1.4 The current study

This study attempted to address the above-mentioned research gaps by investigating the reciprocal relationships between parental anxiety and child anxiety in a four-wave longitudinal Chinese parent-child sample using RI-CLPM. Besides, gender and age differences in these relationships were further explored. Based on existing theories and literature, we hypothesized that a bidirectional relationship would exist between parental anxiety and child anxiety (Hypothesis 1). Additionally, based on the existing studies (18, 36), we hypothesized that compared with boys, parental anxiety would have a stronger effect on girls’ anxiety (Hypothesis 2a); boys’ anxiety would be more likely to trigger parents’ anxiety than girls’ anxiety (Hypothesis 2b). Regarding developmental differences, we anticipated that parental anxiety would exert a stronger influence on children’s anxiety than adolescents’ anxiety (Hypothesis 3a). Besides, compared to adolescents’ anxiety, children’s anxiety would have a greater impact on parental anxiety (Hypothesis 3b).

## 2 Methods

### 2.1 Participants

Data for the present study were collected at four-time points in Chengdu, Sichuan Province, China. Informed consent was obtained from the schools and guardians of the students prior to administering the questionnaires. At the beginning of data collection wave, school, parental and student consent was sought and the participants were reminded that participation was voluntary and they had the right to withdraw at any time. The Positive Youth Development Program collected the data in the 2019, the current study included the data collected from Time 1, Time 2, Time 3 and Time 4. The inclusion criteria as follows (1): parents and students who completed the questionnaire in all four time points of the study (2); the missing rate of less than 50% (3); missing data of less than 10% for the parent anxiety and child anxiety variables. The total response rate was 14,462, with 6,117 participants meeting the inclusion criteria for analysis. Of the children, 3,122 (51.00%) were boys and 2,995 (49.00%) were girls, 2,379 (38.90%) were children, and 3,738 (61.10%) were adolescents, with an average age of 10.32 years (*SD* = 2.14). Of the parents, 2,264 (37.01%) were fathers with an average age of 39.31 years (*SD* = 6.18), and 3,853 (62.99%) were mothers, with an average age of 36.80 years (*SD* = 5.84).

### 2.2 Measures

#### 2.2.1 Parental anxiety

Parental anxiety was measured using the Self-Rating Anxiety Scale (SAS), developed by Zung (60) and translated into Chinese by Wang and Chi (61). This questionnaire consists of 20 items, each rated on a 4-point Likert scale, with higher scores reflecting greater levels of anxiety. The SAS has demonstrated strong reliability and validity in Chinese samples (62). In this study, the SAS indicated excellent internal consistency across all four time points, as evidenced by Cronbach’s alpha coefficients of 0.793(T1), 0.862(T2), 0.817(T3), and 0.795(T4).

#### 2.2.2 Child and adolescent anxiety

Anxiety was measured using the Screen for Child Anxiety Related Emotional Disorders (SCARED) (63). This 41-item questionnaire is divided into five subscales: somatic symptoms, social phobia, school phobia, separation anxiety, and generalized anxiety. Children and adolescents rated each item on a 3-point Likert scale, with higher scores reflecting greater levels of anxiety. The SCARED has demonstrated strong reliability and validity in Chinese children and adolescents (4). In this study, the SCARED indicated excellent internal consistency across all four time points, as evidenced by Cronbach’s alpha coefficients of 0.945(T1), 0.953(T2), 0.954(T3), and 0.943(T4).

### 2.3 Data analysis

SPSS 24 was used to conduct correlation analyses, reliability test, and intraclass correlation coefficient (ICC) analyses. Then, Mplus 8.3 was used to perform random intercept cross-lagged regression analysis to examine the bidirectional relationship between parental anxiety and child anxiety in the four measurements, and further explore the possible gender and age differences in this relationship. Furthermore, SPSS 24.0 was used to perform multiple imputations for missing data, with five imputations performed (57).

## 3 Results

### 3.1 ICC analysis

The descriptive statistics and correlation analyses for this study were conducted using SPSS 24.0, while ICC analysis in Mplus 8.3. For parental anxiety, the ICC of 0.711 indicates that 71.1% of the variance across four repeated measures is due to stable, between-person differences, with the remaining 28.9% stemming from within-person variability. On the other hand, child anxiety presented an ICC of 0.588, revealing an identical distribution of variance-58.8% between individuals and 41.2% within individuals across four time points. These ICC values highlight the necessity of exploring both inter-individual and intra-individual dynamics in parental and child anxiety, as these components reflect the degree to which anxiety levels are influenced by stable individual traits or between individual factors.

### 3.2 Descriptive analyses

**Table 1** presents the means and standard deviations of the study variables across the four time points.

**Table 1:** Descriptive statistics and correlation analysis for mains variables.

Variables	M	SD	1	2	3	4	5	6	7	8
1. T1 PA	1.63	.30	1							
2. T2 PA	1.62	.29	.35^**^	1						
3. T3 PA	1.62	.28	.30^**^	.38^**^	1					
4. T4 PA	1.60	.23	.11^**^	.11^**^	.13^**^	1				
5. T1 CA	.41	.36	.21^**^	.12^**^	.10^**^	.13^**^	1			
6. T2 CA	.36	.36	.19^**^	.17^**^	.12^**^	.03^*^	.49^**^	1		
7. T3 CA	.37	.36	.15^**^	.13^**^	.21^**^	.05^**^	.37^**^	.46^**^	1	
8. T4 CA	.31	.26	.06^**^	.03^*^	.05^**^	.17^**^	.12^**^	.15^**^	.18^**^	1

PA, parental anxiety; CA, child anxiety; T1, Time 1; T2, Time 2; T3, Time 3; T4, Time 4; ^*^ *p* < 0.05, ^**^ *p* < 0.01.

### 3.3 Results of random intercept cross-lagged panel models

**Figure 1:**
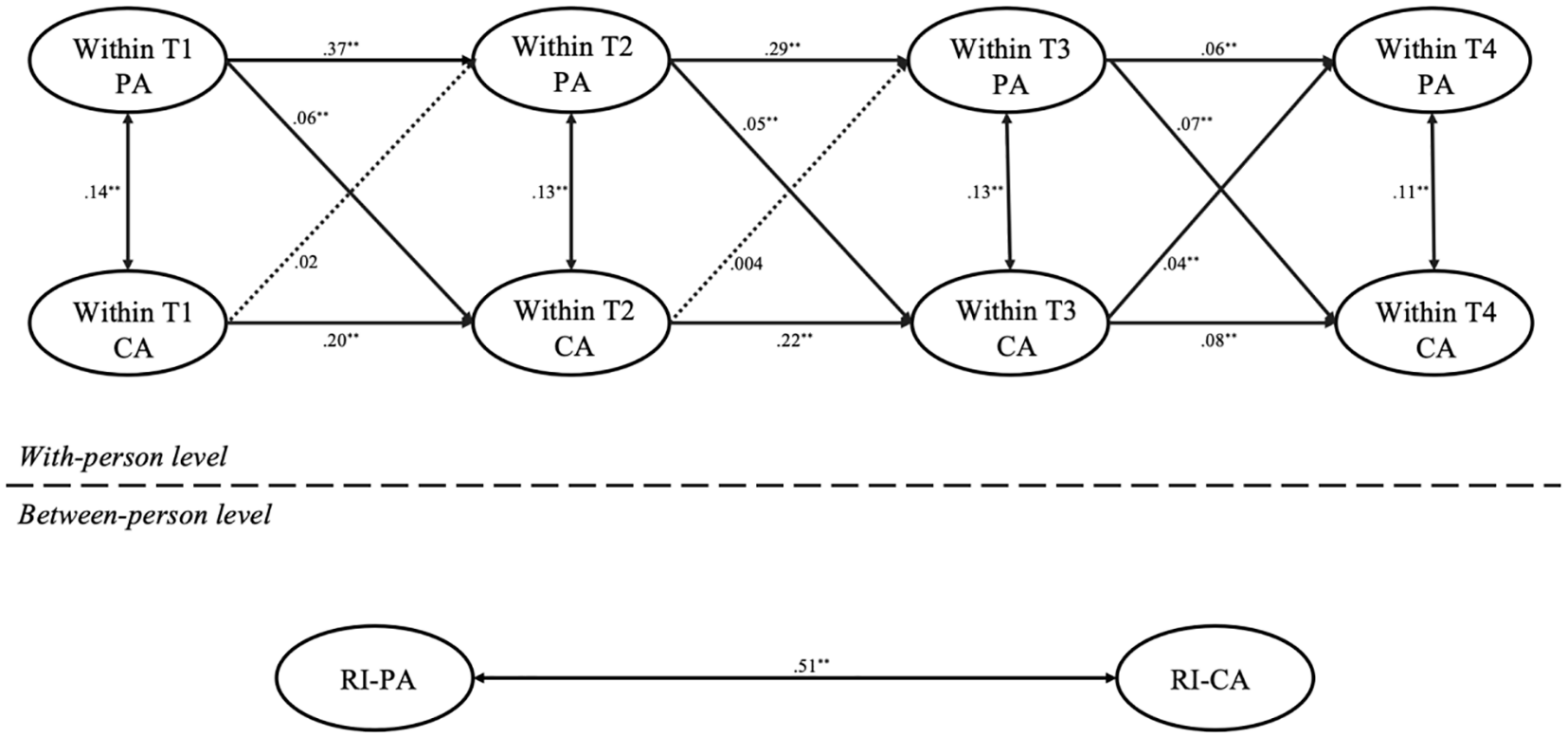
RI-CLPM for the relationship between parental and child anxiety across the four time points in all samples. PA, parental anxiety; CA, child anxiety; T1, Time 1; T2, Time 2; T3, Time 3; T4, Time 4; ^*^ *p* <.05, ^**^ *p* <.01. Dotted lines represent nonsignificant paths.

**Figure 1** presents the model fit and comparison results for the RI-CLPM analysis of parental anxiety and child anxiety. The model fit indices were good: *χ*² = 164.33, *df* = 11, *χ*²/*df* = 14.93, *p* <.001; CFI = .97, TLI = .93, RMSEA = .05, SRMR = .03. The random intercept cross-lagged analysis revealed several observations. First, at the inter-individual level, there was a significant positive correlation between the random intercepts of parental anxiety and child anxiety (*r* = .51, *p* < 0.001), indicating a strong inter-individual relationship between the “traits” of parental and child anxiety. Second, at the intra-individual level, the autoregressive paths showed that parental anxiety at T1 predicted parental anxiety at T2 (*b* = .37, *p* <.01), T2 predicted T3 (*b* = .29, *p* <.01), and T3 predicted T4 (*b* = .06, *p* <.01). Similarly, T1 child anxiety predicted T2 child anxiety (*b* = .20, *p* <.01), T2 predicted T3 (*b* = .22, *p* <.01), and T3 predicted T4 (*b* = .08, *p* <.01). Third, in the cross-lagged paths, T1 parental anxiety predicted T2 child anxiety (*b* = .06, *p* <.01), T2 parental anxiety predicted T3 child anxiety (*b* = .05, *p* <.01), and T3 parental anxiety predicted T4 child anxiety (*b* = .07, *p* <.01). However, T1 child anxiety did not predict T2 parental anxiety (*b* = .02, *p* >.05), nor did T2 predict T3 parental anxiety (*b* = .004, *p* >.05). Notable, T3 child anxiety predicted T4 parental anxiety (*b* = .04, *p* <.01).

### 3.4 Differences between boys and girls

**Figure 2:**
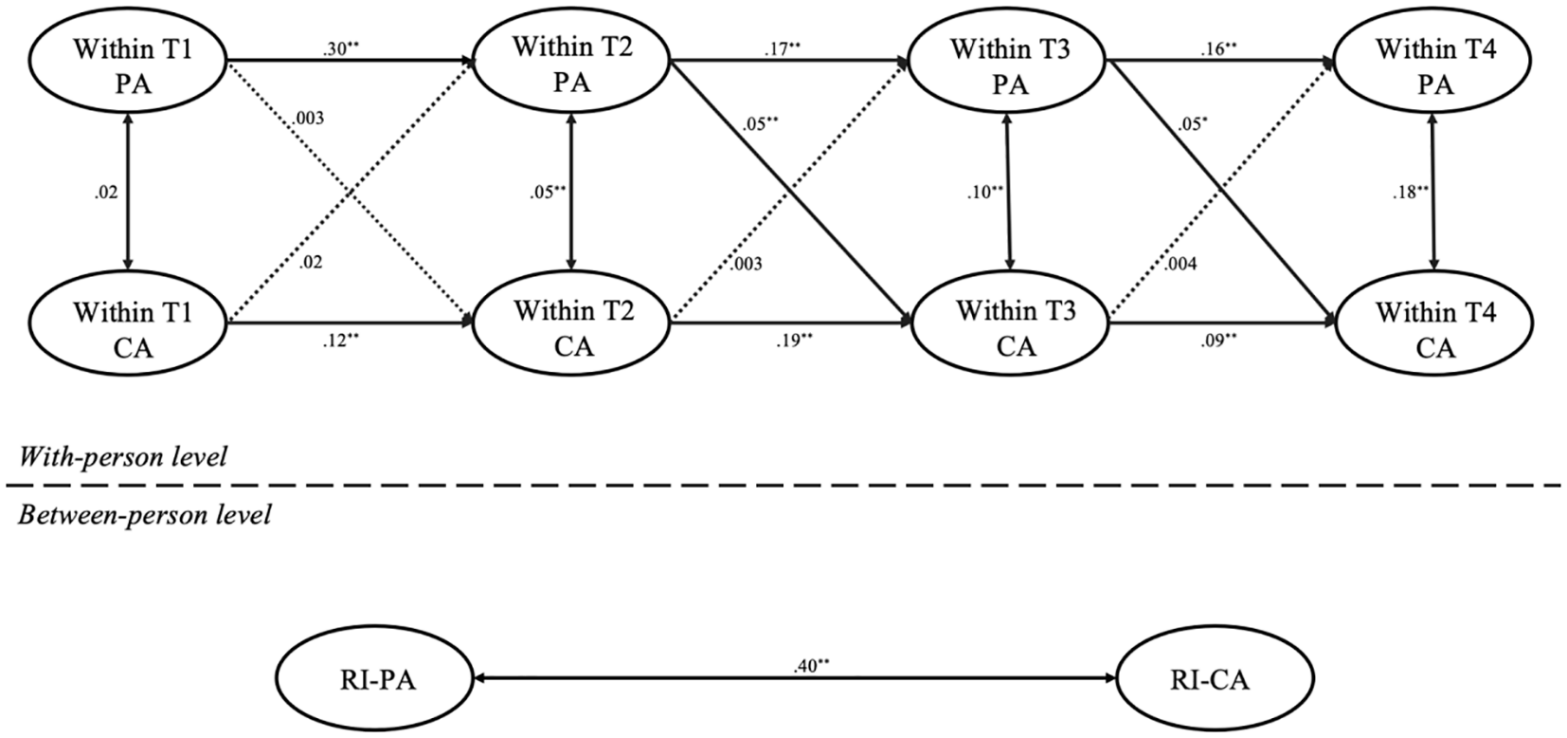
RI-CLPM for the relationship between parental and child anxiety across the four time points in the boys’ sample. PA, parental anxiety; CA, child anxiety; T1, Time 1; T2, Time 2; T3, Time 3; T4, Time 4; ^*^ *p* <.05, ^**^ *p* <.01. Dotted lines represent nonsignificant paths.

**Figure 3:**
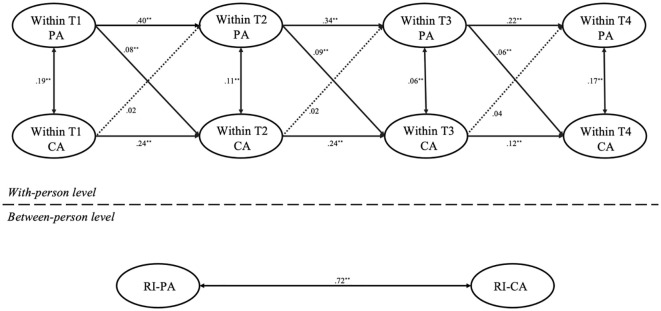
RI-CLPM for the relationship between parental and child anxiety across the four time points in the girls’ sample. PA, parental anxiety; CA, child anxiety; T1, Time 1; T2, Time 2; T3, Time 3; T4, Time 4; ^*^ *p* <.05, ^**^ *p* <.01. Dotted lines represent nonsignificant paths.

First, we used Mplus 8.3 to build a multiple-group RI-CLPM without constraints across the different gender groups which showed a good fit: *χ*² = 155.64, *df* = 18, *χ*²/*df* = 8.65, *p* <.001; CFI = .98, TLI = .94, RMSEA = .05, SRMR = .03. Next, we constructed a constrained lagged-parameters model based on gender. This model also showed good fit: *χ*² = 711.06, *df* = 36, *χ*²/*df* = 19.75, *p* <.001; CFI = .96, TLI = .93, RMSEA = .07, SRMR = .06. The results of the two models are displayed in **Figures 2**, **3**. The chi-square difference test between the two nested models yielded significant results, Δ*χ* ^2^ (18) = 30.86, *p* < 0.001, indicating notable differences in model fit across gender groups. Specifically, differences were observed in the pathway where T1 parental anxiety predicting T2 child anxiety. A Wald test (*t* = 2.64, *p* <.01) showed that T1 parental anxiety predicted T2 child anxiety in girls (*b* = .08, *p* <.01), but not in boys (*b* = .003, *p* > 0.05). Similarly, T2 parental anxiety predicting T3 child anxiety was significant in girls (*b* = .09, *p* <.001), and a smaller effect for boys (*b* = .05, *p* <.01), with a Wald test result (*t* = 2.04, *p* <.05). For the pathway where T3 parental anxiety predicting T4 child anxiety, a Wald test (*t* = 3.07, *p* <.01) revealed a significant effect for girls (*b* = .06, *p* <.01) and a smaller effect for boys (*b* = .05, *p* <.05). However, no significant gender differences were found in the predictive pathways of child’s anxiety on parental anxiety.

### 3.5 Differences between children and adolescents

**Figure 4:**
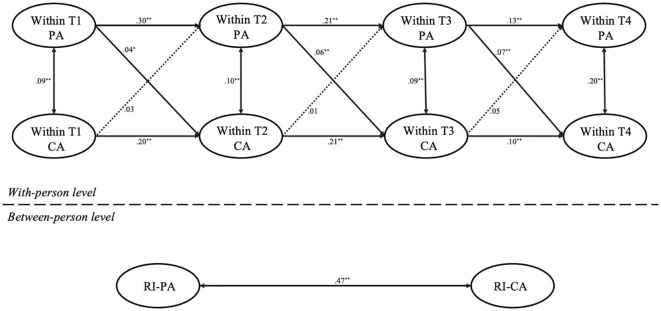
RI-CLPM for the relationship between parental and child anxiety across the four time points in the children’s sample. PA, parental anxiety; CA, child anxiety; T1, Time 1; T2, Time 2; T3, Time 3; T4, Time 4; ^*^ *p* <.05, ^**^ *p* <.01. Dotted lines represent nonsignificant paths.

**Figure 5:**
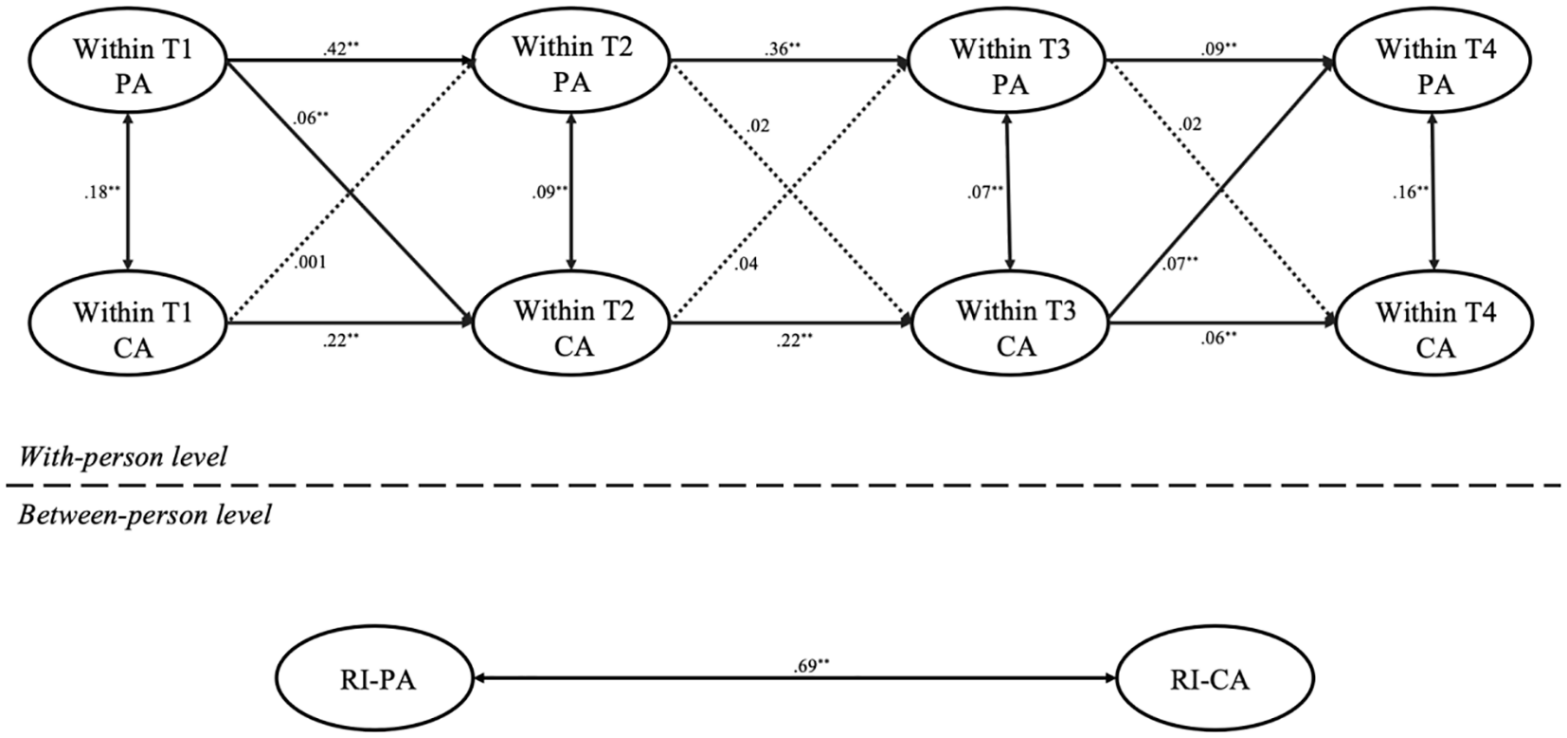
RI-CLPM for the relationship between parental and child anxiety across the four time points in the adolescents’ sample. PA, parental anxiety; CA, child anxiety; T1, Time 1; T2, Time 2; T3, Time 3; T4, Time 4; ^*^ *p* <.05, ^**^ *p* <.01. Dotted lines represent nonsignificant paths.

We used Mplus 8.3 to build a multiple-group RI-CLPM without constraints across the different age groups was fitted: *χ* ^2^ = 143.52, *df* = 18, *χ* ^2^ */df* = 7.97, *p* <.001; CFI = .98, TLI = .94, RMSEA = .05, SRMR = .03. Then, we constructed a constrained lagged-parameters model. This model also showed good fit: *χ* ^2^ = 542.98, *df* = 36, *χ* ^2^ */df* = 15.08, *p* <.001; CFI = .97, TLI = .95, RMSEA = .07, SRMR = .06. The results of the two models are displayed in **Figures 4**, **5**. The chi-square difference test between the two nested models yielded a significant result, Δ*χ*² (18) = 22.19, *p* <.01, indicating that the model fit differs significantly between the child and adolescent groups. Specifically, differences were observed in the pathway of T2 parental anxiety predicting T3 child anxiety. A Wald test (*t* = 2.93, *p* <.01) showed that T2 parental anxiety significantly predicted T3 child anxiety in children (*b* = .06, *p* <.01), but not among adolescents (*b* = .02, *p* >.05). Similarly, T3 parental anxiety significantly predicted T4 child anxiety in children (*b* = 0.07, *p* < 0.001), but not in adolescents (*b* = .02, *p* >.05) (Wald test: *t* = 3.19, *p* <.01). As for the pathways where T3 child anxiety predicting T4 parental anxiety, although the Wald test did not reveal significant differences, the pathway showed distinct results between children and adolescents, with T3 child anxiety predicting T4 parental anxiety among adolescents (*b* = .07, *p* <.01), but not among children (*b* = .05, *p* >.05).

## 4 Discussion

This study employs a four-wave longitudinal design and uses a random intercept cross-lagged panel model to investigate the reciprocal relationship between parental and child anxiety. Additionally, the study explores gender and age-related differences, providing a deeper understanding of how these factors may influence the anxiety dynamics between parents and children.

### 4.1 Reciprocal associations between parental anxiety and child anxiety

The results indicated that parental anxiety at T_n consistently predicted child anxiety at T_n+1 from T1 to T4, supporting the “parent effect” model. Furthermore, child anxiety at T3 was found to significantly predict parental anxiety at T4, supporting the “reciprocal effects” model, emphasizing a bidirectional interaction during this particular period. Hypothesis 1 is partially supported. The result also supports attachment theory, which suggests that parental anxiety can lead to negative psychological representations of the self and others, further inducing negative emotions and behaviors such as anxiety (12, 13).

The present findings highlight that parental anxiety influences children’s anxiety all time, which supports previous studies (16). There are several explanations. First, children develop similar emotional responses and coping strategies by observing how their parents handle stress and threats in daily life (64). The emotional reactions displayed by parents are easily detected by children, who may adopt their parents’ anxious response patterns through imitation (38). Second, parental anxiety can lead to a decline in parenting quality, which, in turn, impacts children’s emotional development. Anxious parents are more sensitive to parenting stress (65) and often exhibit over-controlling or insufficiently warm parenting behaviors (66). Such behaviors can hinder children’s ability to independently solve problems and increase their feelings of uncertainty and anxiety in novel situations. Third, parental anxiety can affect children’s attachment security. Research indicates that anxious parents may struggle to provide stable emotional support, thereby fostering insecure attachment within the parent-child relationship (67). This insecure attachment shapes children’s expectations of social interactions through “Internal working models,” making them more prone to feelings of rejection or neglect during interactions (11, 68). As a result, these children may become more susceptible to anxiety during childhood and adolescence. Moreover, parental anxiety conveys a perception of the environment as threatening (17). Studies have found that parents’ interpretations of environmental threats significantly influence children’s cognitive processing of ambiguous cues (69). For instance, when parents display excessive worry or anxiety about routine events, children are more likely to interpret similar situations as threats, resulting in increased anxiety levels (70).

Additionally, there was a bidirectional interaction between parental and child anxiety during the interval from T3 to T4, which supports the “reciprocal effects” model. According to previous research, children’s emotional adjustment issues may indirectly increase the risk of parental anxiety by increasing the level of parents’ external daily stresses (71). In particular, the period from T3 to T4 may be a time of developmental transition for children. They are more likely to experience increased emotional and behavior issues, such as depression, behavioural withdrawal, or academic issues (72, 73). Therefore, adolescent problem behaviors may increase parental mental stress, which in turn increases parental anxiety.

### 4.2 Gender differences in the relationships

Our findings revealed that the influence of parental anxiety on girls’ anxiety was more significant than on boys’, supporting previous studies (18). This result supports Hypothesis 2a. Gender differences in emotional regulation may explain the result. Studies have found that women are more sensitive to emotions (74), particularly negative emotions. This makes women more susceptible to negative emotions, such as parental anxiety (75). In addition, compared with boys, girls often face greater challenges in regulating negative emotions, which increases their likelihood of developing emotional disorders, such as anxiety (76). Finally, according to the Response Style Theory (77), women’s propensity toward ruminative thinking makes it more challenging to shift their attention and intensifies their emotional responses (78). This persistent focus on negative emotions can lead to negative self-schemas, which increase anxiety (79). Hence, when perceiving parents’ anxiety, girls are more likely to feel uneasy and threatened by parents’ anxious emotions due to their high sensitivity to negative emotions and tendency to ruminate. This perception triggers their overreaction to emotions and constant inner rumination, thus exacerbating their experience of anxiety.

In addition, no significant gender differences were found in the predictive pathways of child’s anxiety on parental anxiety, Hypothesis 2b wasn’t supported. Studies have indicated that in modern families, parents generally show similar levels of concern for both boys and girls, which means gender differences or children’s gender-differentiated emotion won’t elicit differential parental treatment (80). When children experience anxiety, parents often respond with worry and stress. Regardless of the child’s gender, their anxiety will lead to the same anxious reactions.

### 4.3 Developmental differences between children and adolescents

This study further explored the developmental differences between children and adolescents, revealing that parental anxiety had a significantly greater impact on children’s anxiety, supporting Hypothesis 3a. This finding may be closely related to the differences in psychological characteristics between children and adolescents at different stages. First, children’s emotional processing abilities have not fully developed, making them more reliant on parents’ emotional states (40). Therefore, children are more likely to be influenced through emotional contagion mechanisms and directly imitating parents’ anxious reactions when they perceive parental anxiety (38). Additionally, as adolescents grow, they shift from relying on their parents to seeking emotional support from peers or other external resources (81). This shift not only decreases adolescents’ sensitivity to their parents’ emotions, but also buffers the emotional pressure from the family through peer interactions (82). Therefore, the impact of parental anxiety on adolescent anxiety is relatively weaker, reflecting significant age-related differences in emotional transmission.

The study also found that adolescent anxiety at T3 significantly predicted parental anxiety at T4 which partially supports Hypothesis 3b. There are several factors that may explain why adolescent anxiety can lead to parental anxiety. First, research has shown that adolescent anxiety is often accompanied by internalizing or externalizing behavioral problems, such as withdrawal, social difficulties, impulsivity, quarreling, and rule breaking (73). These problem behaviors can damage the parent-child relationship and cause additional stress for parents, which in turn increases their anxiety levels. In addition, childhood anxiety is often accompanied by poor academic performance, especially in academics, prompting parents to worry about their growth and future (83). As a result, parents are more concerned about whether their children will be able to enter high school in the future, which makes it easier for them to become more anxious.

### 4.4 Theoretical and practical implications

This study offers valuable insights into the dynamics between parental and child anxiety, contributing to the field in several ways. Firstly, it emphasizes the importance of adopting a dynamic perspective on the relationship between parental and child anxiety. The research results found the applicability of the “parental effect” model and the “reciprocal effect” model at different developmental stages, emphasizing that the relationship between parental anxiety and child anxiety is dynamic, providing a new theoretical perspective for understanding the intergenerational transmission of parent-child emotions. Secondly, this study conducted in a non-Western context, demonstrates that the patterns observed in the “parental-child effects” model is applicable within a Chinese cultural context, extending the relevance of these theories to Chinese context. Overall, this research advances theoretical understanding of anxiety transmission within families by integrating a bidirectional, culturally inclusive, and developmentally sensitive approach.

Also, the findings suggest several key implications for weakening both parental and children’s anxiety. Firstly, we should build routine anxiety screenings in communities and provide emotional management courses for parents to reduce anxiety, such as deep breathing, mindfulness meditation, and other emotion regulation techniques. Secondly, there should be a focus on anxiety in girls and young children, with targeted interventions for girls and young children to help them develop emotion regulation skills. Thirdly, studies indicate that positive youth development programs can effectively alleviate negative emotions such as depression in adolescents (84, 85). Therefore, the education sector can set up a multi-dimensional intervention system involving schools, families and the community to implement positive child and youth development programs.

### 4.5 Limitations and future directions

This study has several limitations. First, the study focused on general anxiety without exploring different types of anxiety, such as trait anxiety, state anxiety, and parents’ educational anxiety. For instance, a study using parents and children’ data in China found that children’s education (such as educational inputs) positively predicted parents’ educational anxiety (86). Future research should further explore how individual experiences and broader social context shape different types of parent anxiety, providing a more comprehensive understanding of intergenerational transmission of different forms of anxiety. Second, this study used self-report questionnaires to collect data, which may be influenced by social desirability bias or recall bias (87). Hence, future studies should utilize multi data source, such as collect teacher evaluations to measure child anxiety. Third, this study primarily examined the direct relationship between parental anxiety and child anxiety, without delving into the underlying mechanisms. Future studies could further explore longitudinal mediators or moderators to examine how parental anxiety influences children’s anxiety through specific psychological processes. Furthermore, we also can collect physiological data (such as cortisol levels), it would offer a more objective measure of anxiety, thereby increasing the study’s validity and reliability. Fourth, RI-CLPM was employed to test the parental anxiety predict child anxiety, only provide directions of the relationship between parental anxiety and child anxiety. Hence, future studies could further construct a latent growth curve model (LGCM) to provide a more precise assessment of developmental trajectories, in order to explore co-development relationships between parental anxiety and child anxiety. Fifth, the study focused only on the relationship between parental anxiety and child anxiety. However, increasing extensive studies have suggested that family factors are important in shaping children behavior (88) such as parental child-rearing gender-role attitudes (89). Besides, research has shown that child gender affects parenting behavior, particularly fathers, such as the amount time of spend with children (90, 91). Therefore, future research should further explore how different parental genders shape the psychological and behavioral development of children of different genders. Finally, in view of the developmental issues in children and adolescents (92), there is a need to consider development of preventive programs (93), preferably with the involvement of the parents.

## 5 Conclusion

This study utilized a four-wave dataset to identify the developmental trajectory of parental anxiety, child anxiety through RI-CLPM, as well as its gender difference and children and adolescents’ difference. The RI-CLPM indicated that parental anxiety significantly predicted subsequent child anxiety, supporting the “parental effect” model. Moreover, child anxiety at T3 significantly predicted parental anxiety at T4, supporting the “reciprocal effect” highlighting a bidirectional interaction at this time point. Moreover, the impact of parental anxiety on girls’ anxiety was significantly greater than on boys, and parental anxiety had a more substantial influence on children than on adolescents. Overall, the present study emphasizes the critical role of parental anxiety in fostering child anxiety at the within- and between-person between-person level, while illustrating the impact of parental anxiety on children’s anxiety of different genders and ages in the Chinese cultural context. This temporal variability suggests that the interaction between parental and child anxiety is not static but evolves dynamically. Therefore, interventions should therefore be dynamic and tailored to the specific gender and stage, addressing the unique needs and challenges of both parents and children anxiety.

## Data Availability

The raw data supporting the conclusions of this article will be made available by the authors, without undue reservation.
